# Prevalence of Oral Potentially Malignant Disorders Among Tobacco Users in Kolkata: A Hospital-Based Study

**DOI:** 10.7759/cureus.72084

**Published:** 2024-10-22

**Authors:** Divya Pandya, Anwesha Banerjee, Arpita Maitra, Rekha Puttanavar, Piyali Datta, Ishan Mukherji

**Affiliations:** 1 Oral Medicine and Radiology, Guru Nanak Institute of Dental Sciences and Research, Kolkata, IND; 2 Oral Medicine and Radiology, D.Y. Patil University School of Dentistry, Navi Mumbai, IND; 3 Pedodontics and Preventive Dentistry, Guru Nanak Institute of Dental Sciences and Research, Kolkata, IND; 4 Public Health Dentistry, Guru Nanak Institute of Dental Sciences and Research, Kolkata, IND

**Keywords:** gutka, oral cancer, oral potentially malignant disorder, smokeless, tobacco

## Abstract

Introduction

Tobacco consumption is a significant public health threat worldwide and a looming pandemic. World Health Organization data display that about five million people face premature death per year worldwide due to tobacco use. India is a leading nation among tobacco users in this regard. The aim of the present study was to determine the most prevalent tobacco consumption habit and the oral potentially malignant disorders (OPMDs) found in such users with an assessment of awareness and change in various habits.

Material and methods

A cross-sectional study over a duration of six months was carried out, in which every patient visiting the hospital OPD was assessed and evaluated for tobacco-related habits. A detailed history of the duration and frequency of the habit, the type of tobacco product consumed, and the predominant reason for its consumption among males and females were recorded and evaluated. An oral examination was performed to determine the evidence of OPMDs. Subjects under the age of 13 years and with systemic/metabolic disorders with oral manifestations were excluded from the study. Data obtained were subjected to statistical analysis using Stata Statistical Software, version 13.1 for Windows (released 2013 StataCorp LLC, College Station, TX).

Results

Data revealed a higher prevalence of tobacco consumption in males, with chewing tobacco followed by smoking and a mixed form of habit being predominant. Misri chewing was most common in females. Subjects in the 20-50 years age group were most commonly affected, with tobacco pouch keratosis being the most prevalent lesion followed by leukoplakia, with stress being a major causative factor for tobacco consumption.

Conclusion

The present study promulgated the prevalence of OPMDs in consumers of various kinds of tobacco products, which is highly valuable in the early detection of OPMDs. OPMDs remain unnoticed until advanced stages due to their asymptomatic or mildly symptomatic nature and due to a relative lack of awareness about the deleterious effects of tobacco usage despite an upsurge in tobacco cessation-related content on digital media. Thus, healthcare sector professionals can be appropriately guided to ensure efficient patient care at an early stage.

## Introduction

Oral cancer has emerged as a significant global health concern in the past two decades. As per global health statistics 2020, 377,713 new cases with 177,757 deaths were reported due to oral cancer. Asia reports more than two-thirds of cases of oral cancer, with India alone contributing to one-third of cases globally [1]. Tobacco usage is among the leading cause of oral cancers worldwide. Tobacco-induced oral cancer is the third most common cancer type in South-Central Asia and tops the chart in India. A wide range of oral mucosal potentially malignant disorders, such as leukoplakia, erythroplakia, and oral submucous fibrosis (OSMF), associated with tobacco usage, leads to a potential risk of developing oral cancer [2-4]. Global Adult Tobacco Survey-2 (GATS-2) reported in 2016-2017 that 28.6% of Indian people are tobacco consumers, with 10.7% being smokers and 21.4% consuming smokeless or chewing form of tobacco [2] that India stands as the second largest tobacco consumer and third largest tobacco producer globally [5]. World Health Organisation (WHO) claimed that tobacco-induced mortality rates may exceed 1.5 million in India annually by 2020 [1]. In India, tobacco products are commercially and readily available either as smoking cigarettes, bidi, chutta, chillam, hookah, or in smokeless forms like khaini, zarda, mishri, gul, mawa, and gudaku, which contain hydrocarbons and potent nitrosamines, which are in turn DNA toxic carcinogens [6,7]. Tobacco being a social nuisance, consumers have shifted to non-tobacco products like areca nut [5]. Betel nut chewing bagged the fourth position among the most addictive substances globally, preceded by tobacco, alcohol, and caffeine [1]. In India, due to variable socio-economic status, wide geographical diversity, and multiple ethnic populations, the type of tobacco consumed and its associated oral lesions vary inordinately [8]. The preferences of tobacco consumption also vary with gender and geography, such as betel quid is preferred by females of northeast and southern India, while women and men belonging to eastern, western, and central India prefer khaini and gutka chewing. Thus, to formulate government policies and obtain national data relevant to different states, ethnic groups, and genders in India, it is necessary to collect data from state levels from both urban and rural populations [6]. Oral potentially malignant disorders (OPMDs) and oral cancers result in heavy impairment of quality of life. Primary prevention is the most cost-effective prevention program that can reduce the incidence of OPMDs by controlling risk factors. The majority of the population is unaware of the risks of OPMDs and ways to prevent them [9], especially in rural areas where oral cancer screening gets impeded due to poor awareness, self-neglect, late presentation of lesions, poor health literacy, lack of appropriate health infrastructure needed for early lesion detection, dominance of alternative therapies and unlicensed practitioners, easy availability of tobacco and related products and alcohol, poor socio-economic status with lack of health insurance, social stigma, distance and transportation difficulties with poor referral pathways, resistance to quit the habit, and fear of cancer diagnosis. Our government is constantly formulating policies and programs for early detection of cancers and OPMDs at all levels, which includes the establishment of the Health and Wellness Centre (HWC) under the Ayushman Bharat Programme, the National Oral Health Program, the National Tobacco Control Program, and tobacco cessation centers [10]. Regular and thorough oral visual screening (OVS) is the most reliable and easy method, and it is an urgent need that can contribute significantly to early detection to reduce the incidences of OPMDs, their malignant transformation rate, and morbidity and mortality associated with oral cancers due to their late detection. Hence, more studies are required at the smallest possible level to understand the epidemiology of this destructive condition, and data can be compiled at the national level for the betterment of society [2,9]. Thus, the current study was conducted with the aim of determining the prevalence of OPMDs among tobacco users in a hospital-based sample of the Kolkata population.

## Materials and methods

Study setting and population

An observational questionnaire-based cross-sectional study with a duration of six months was performed at Guru Nanak Institute of Dental Sciences and Research, Kolkata, wherein all the patients visiting the OPD were assessed and quizzed regarding their tobacco-related habits. Patients under the age of 13 years and patients with systemic and/or metabolic disorders with oral manifestations, orofacial trauma, immunocompromised status, and mental instability were excluded from the study. The study was approved by the institutional ethics committee prior to commencement and conducted as per STROBE (Strengthening the Reporting of Observational Studies) guidelines (see Appendix).

Data collection

The self-designed questionnaire was pretested and checked for validity and reliability. Informed consent in all local languages was obtained from all subjects who participated in the study. A detailed history of 300 tobacco (smokeless and smoking forms) consuming patients regarding the duration and frequency of the tobacco consumption habit along with its type and predominant reason for its consumption among males and females along with their age were recorded and evaluated for the evidence of OPMDs. Face-to-face interviews were conducted by only one trained and calibrated examiner. A thorough oral clinical examination and OVS were performed to identify the presence of any OPMDs under incandescent light using a sterile mouth mirror by experienced oral medicine personnel.

Statistical analysis

The entire data from this study were entered in a master chart, and then data analysis was done using Stata Statistical Software, version 13.1 for Windows (released 2013 StataCorp LLC, College Station, TX). Demographic measurements were summarized descriptively by age groups and gender. Summary statics were provided for all parameters, including mean and standard deviation (SD) 95% confidence interval for continuous variables, and categorical variables were presented with frequency and percentage.

## Results

Demography-related results showed that out of 300 study patients, males (69.3%, n = 208) were more than females (30.7%, n = 92).

**Figure 1:**
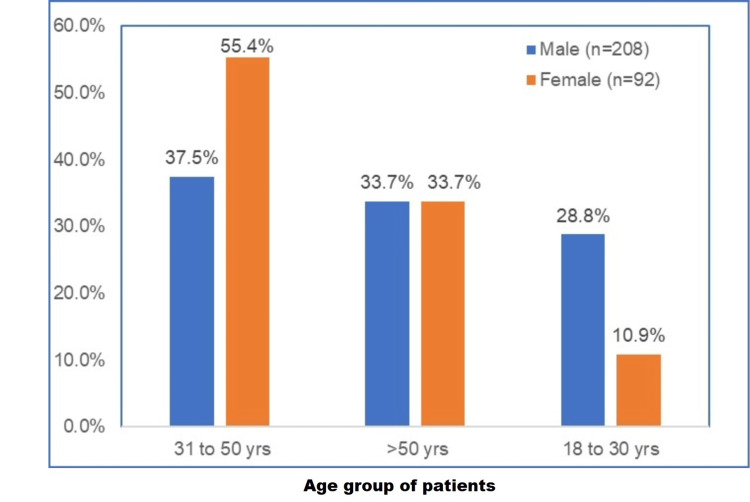
Distribution of age group of patients n = 300, males (n = 208), females (n = 92) % of n (separate for males and females)

Males were predominant tobacco users among the 18-30 age group (28.8%, n = 60), females were predominant among the 31-50 age group (55.4%, n = 51), and percentage of male (33.7%, n = 70) and female (33.7%, n = 31) users were same in age group above 50 years (Figure 1).

**Figure 2:**
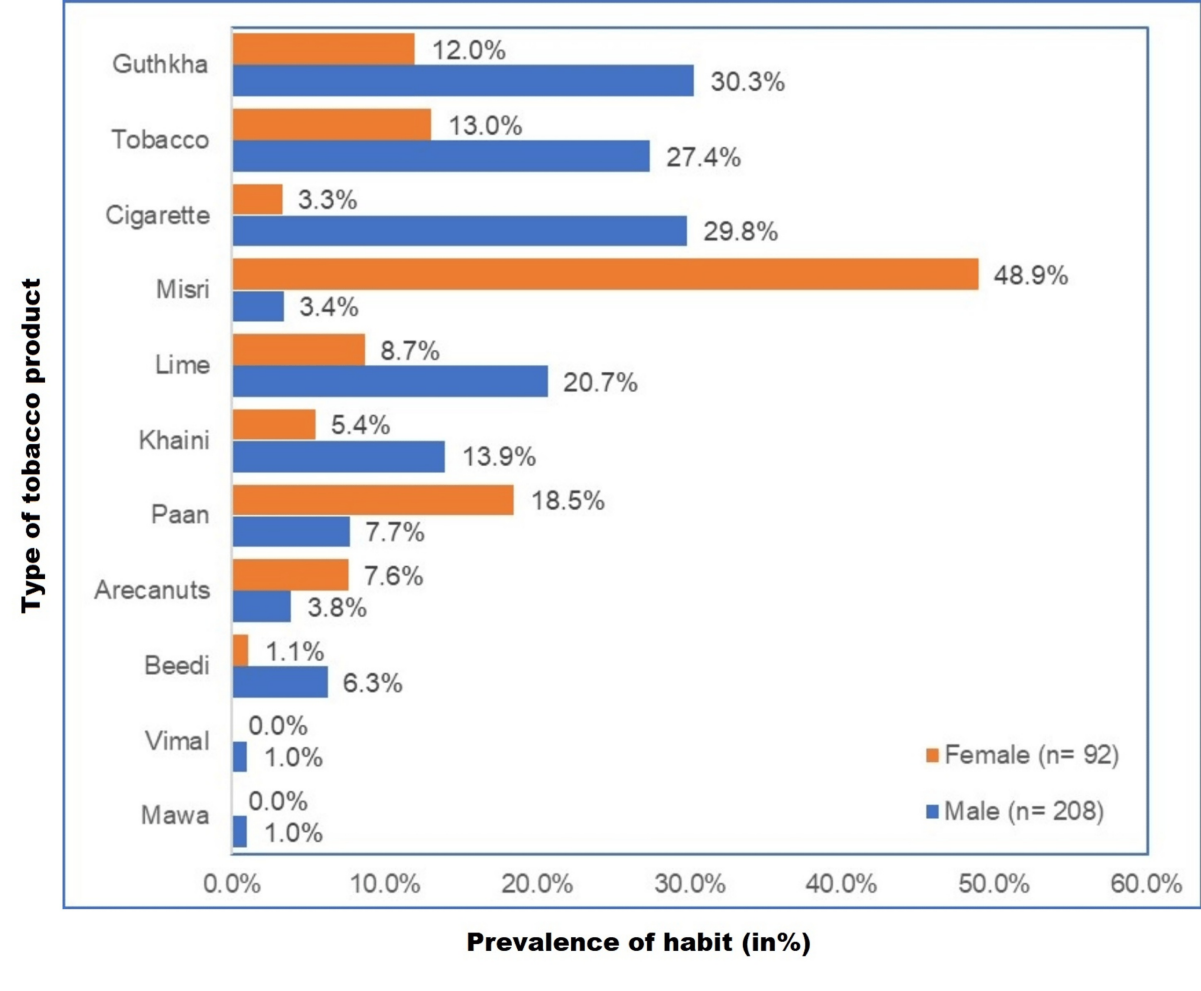
Prevalence of type of tobacco habit n = 300, males (n = 208), females (n = 92) % of n (separate for males and females)

The most predominant form of tobacco consumption in male patients was gutka chewing (30.3%, n = 63), followed by cigarette smoking (29.8%, n = 62) and plain tobacco chewing (27.4%, n = 57), and in females, misri chewing (48.9%, n = 45) was the most predominant type of tobacco product followed by paan chewing (18.5%, n = 17) and plain tobacco (13%, n = 12) and gutka chewing (12%, n = 11) (Figure 2).

**Figure 3:**
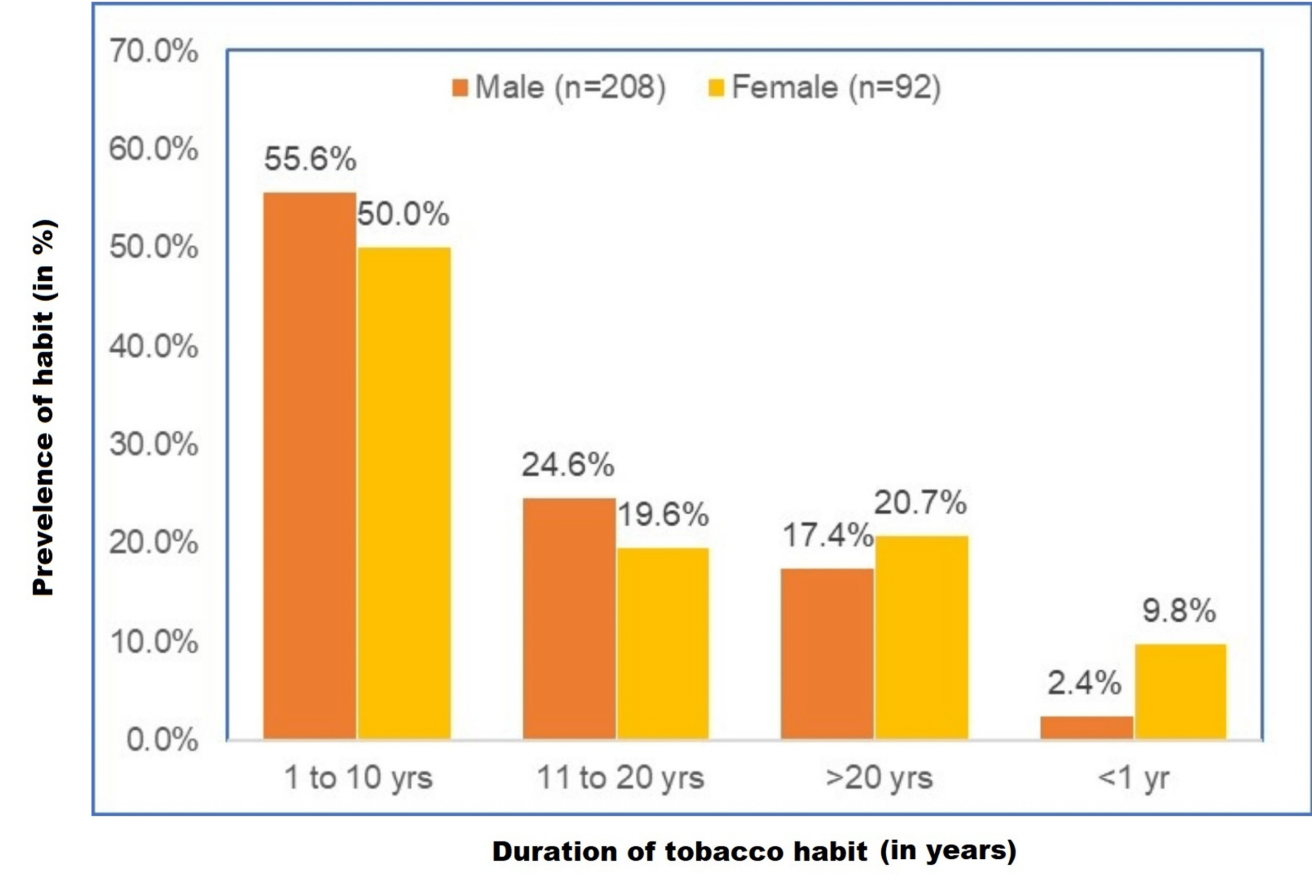
Distribution of duration of tobacco consumption n = 300, males (n = 208), females (n = 92) % of n (separate for males and females)

Our study showed that males were predominant, within one to 10 years (55.6%, n = 116) and 11-20 years (24.6%, n = 55) history of duration of consumption of tobacco over females, but in the history of duration of tobacco consumption within more than 20 years (20.7%, n = 19) and less than one year (9.8%, n = 9), females were more predominant (Figure 3).

**Figure 4:**
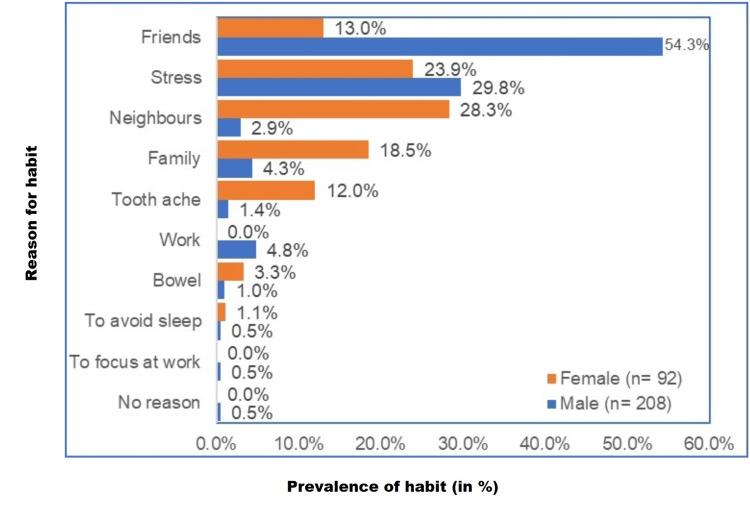
Distribution of reasons for tobacco use n = 300, males (n = 208), females (n = 92) % of n (separate for males and females)

The most common reason for tobacco consumption in males was friends (54.3%, n = 113), followed by stress (29.8%, n = 62), whereas in female subjects, neighbors (28.3%, n = 26) were the most common reason followed by stress (23.9%, n = 22), family (18.5%, n = 17), and friends (13%, n = 12) (Figure 4).

**Figure 5:**
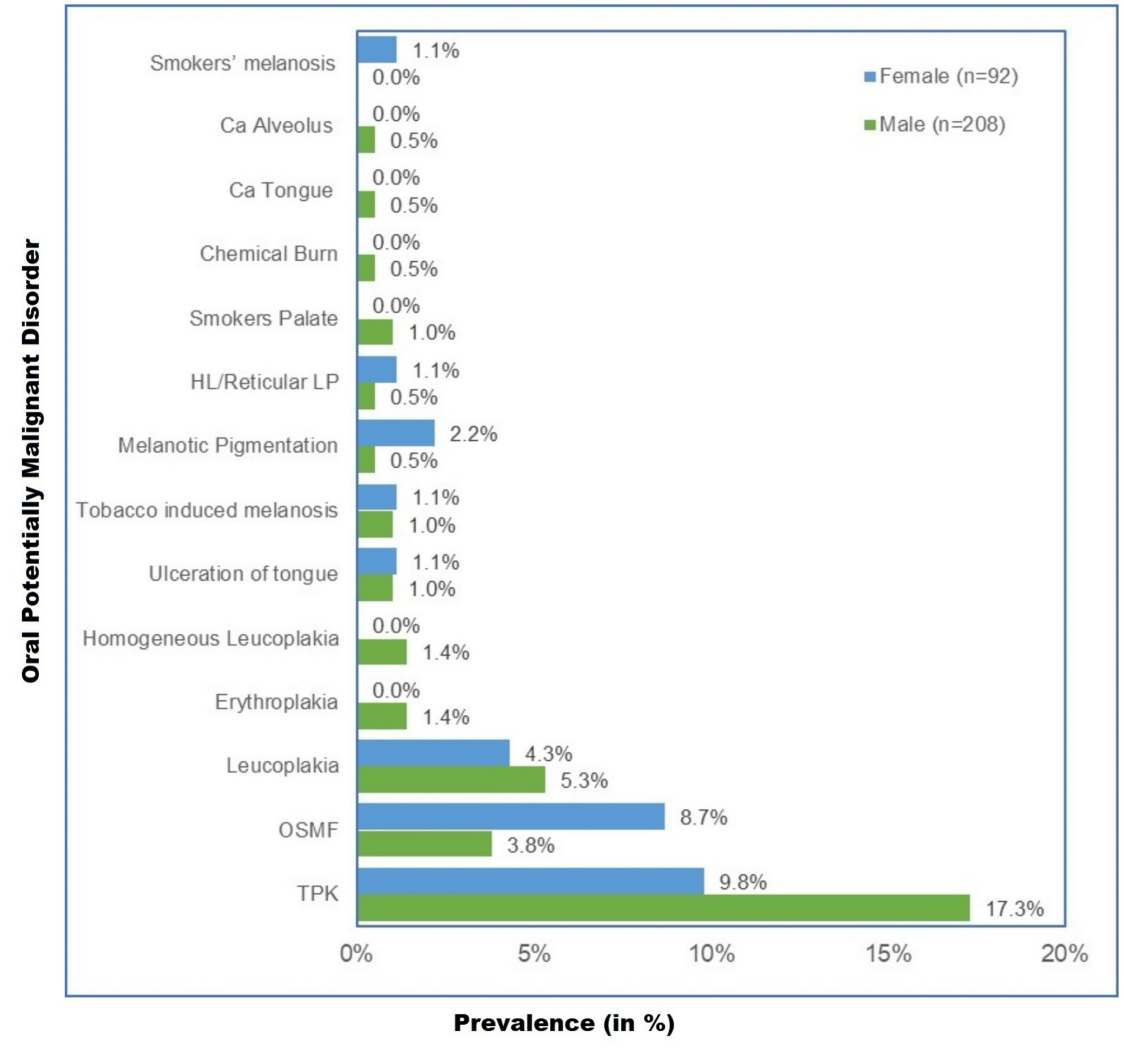
Sex-wise prevalence of oral lesions among tobacco users n = 300, males (n = 208), females (n = 92) % of n (separate for males and females)

In both males (17.3%, n = 48) and females (9.8%, n = 9), tobacco pouch keratosis (TPK) was the most predominant lesion, followed by leukoplakia in males (5.3%, n = 11) and OSMF in females (8.7%, n = 8) (Figure 5).

**Figure 6:**
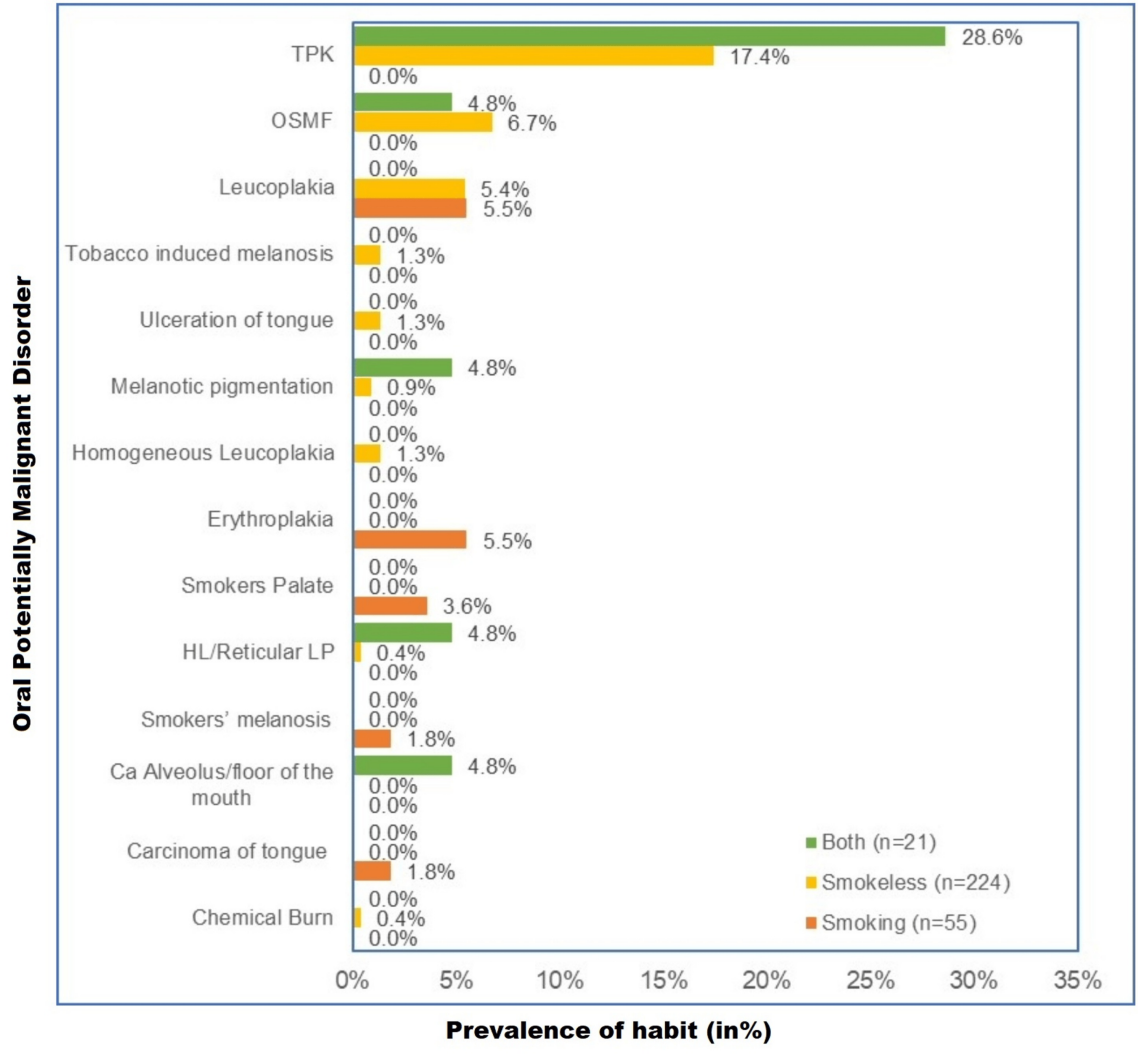
Habit-wise prevalence of oral lesions among tobacco users n = 300, both smoking and smokeless (n = 21), smokeless only (n = 224), smoking only (n = 55) % of n (separate for both smoking and smokeless, smokeless only and smoking only)

In TPK, both forms of tobacco were predominant (28.6%, n = 6), followed by the smokeless form (17.4%, n = 39). In OSMF, smokeless form was more common (6.7%, n = 15). In leukoplakia, smoking was the main cause (5.5%, n = 3). In erythroplakia (5.5%, n = 3) and in smokers’ palate (3.6%, n = 2), the smoking form of tobacco was more predominant (Figure 6).

## Discussion

The high prevalence of OPMDs and oral cancer in India with variation in distribution is due to multiple geographical and ethnic differences, such as tobacco or areca nut or betel quid chewing, which is popular in developing countries, but cigarette smoking with alcohol consumption is common in developed countries. Due to the remarkable variation in the prevalence rate of oral habits among different states in India, it contributes to the prevalence of different OPMDs in different states and groups of people [11]. According to a survey by GATS 2017, in West Bengal, 21.3% of total adults smoked tobacco, and 21.9% consumed a smokeless form of tobacco. National Family Health Survey-4 (NFHS-4) documented that in West Bengal, smoking form of tobacco is more popular in urban areas, and chewing tobacco dominates the rural areas [12].

Our study showed that there was increased male prevalence (69.3%) than females. Male users were more in the 18-30 years group (28.8%), and females were more in the 31-50 years group (55.4%). Our study revealed that gutka chewing was the most common tobacco usage form in males (30.3%), followed by cigarette smoking (29.8%) and plain tobacco chewing (27.4%), and misri consumption was predominant in females (48.9%), followed by pan chewing (18.5%) and plain tobacco (13%) and gutka chewing (12%) thus explaining that in both the genders smokeless form of tobacco was more common than smoking. Our study revealed that TPK (15%) was the most prevalent lesion evident, followed by OSMF (5.3%) and leukoplakia (5%) in both genders.

A similar hospital-based study was conducted in Kolkata in 2013, which showed that males with smokeless tobacco or mixed habits pose a higher risk of developing oral cancer, while areca nut chewing is the predominant threat in females for the development of oral cancers [11].

A study conducted in surrounding districts of Kolkata showed similar results wherein males were predominant tobacco users (78.5%) than females (21.5%). However, this study revealed contradictory results from our study and stated that smoking was more common (26.2%) in their study population, followed by chewable tobacco (20.32%), and pan with areca nut was more predominant chewable tobacco form (6.14%) followed by khaini (5.35%) and mixed habit (2.40%). Contrary to our study, leukoplakia was the most predominant lesion (3.2%) in this study, followed by smoker’s melanosis (2.4%), tobacco-induced keratosis (2.1%), smokers’ palate (1.3%) and OSMF (1.3%) [12].

A prevalence study conducted in Assam found similar results to our study and reported that male predominance (64.6%) was evident among tobacco users more than females (35.4%). Here, the majority of both genders were found among the age group of 41-50 years, and chewing tobacco, predominantly areca nut (9.9%), was more common than smoking (3.46%). Leukoplakia was the most predominant lesion (22.7%), followed by erythroplakia (94.46%), contrary to our results [1].

A community-based cross-sectional study conducted in Vellore reported female predominance among tobacco users (62.2%), and smoking tobacco (59.1%) was a more prevalent habit than smokeless tobacco (38.6%), with smokers’ palate (60.1%) being the most prevalent oral lesion contrary to our results [2].

Two different comparative studies among the socio-culturally different female population of West Bengal were conducted and reported a significant association between smokeless tobacco and less educated females [6], with females from North Bengal being predominant tobacco chewers with a relatively higher prevalence of habit-related oral lesions than South Bengal [5] similar to our study where in females smokeless form of tobacco consumption was more common (39.7%).

A prevalence study conducted in Udupi, Karnataka, showed similar results as that of our study and reported that OPMDs were more prevalent in younger individuals, males, and with smokeless forms of tobacco [13]. In the state of Rajasthan, two prevalence and cross-sectional studies were conducted and showed that smoking was more prevalent than smokeless form of tobacco [14], and smokers’ palate, TPK, and leukoplakia were commonly noted mucosal lesions [15].

Our study showed that both smoking and smokeless forms of tobacco are prevalent in age groups 18-30 years; however, among the other age groups, smokeless forms of tobacco were more common. In 2021, a prevalence study was conducted in a rural community in West Bengal and reported that the mean age of tobacco users was 53.74 years, and chewing tobacco (64.5%) was the most common form, similar to our study [16]. A cross-sectional study with almost similar results as our study was performed in Chhattisgarh, which revealed that the smokeless form of tobacco (54.6%) was the most predominant among Beedi rolling workers and leukoplakia (27.6%) was the most prevalent lesion, followed by OSMF (13.5%) [7]. Research was conducted to determine the prevalence and risk factors for OPMDs in the Indian population, which revealed that the overall prevalence of OPMDs was 13.7%, with OSMF being the most common lesion (8.06%) [9].

In the present study, 52.4% of male patients and 25% of female patients have attempted to quit the habit. A similar study was done in Uttar Pradesh in 2019, which also concluded that smokeless tobacco was more prevalent (42.6%), with gutka with pan being the most consumed form. Tobacco chewing was highest among the 35- to 44-year-old age group, with 69.3% willing to quit smoking and 78.8% willing to quit tobacco [17].

Our study showed that tobacco consumption in males was majorly influenced by friends (54.3%), followed by stress (29.8%), whereas in female subjects, neighbors (28.3%) were the most common reason, followed by stress (23.9%). A study conducted on Nepalese students in 2021 recorded that peer pressure (62.3%) was the most influential factor for tobacco consumption, followed by experimentation (18.2%) and tobacco commercials (7.8%) [18].

Studies conducted abroad also achieved results that were almost similar to those of studies conducted in India. A study done in Sri Lanka revealed that smokeless form of tobacco (41.1%) was more common, and similarly, areca nut/betel quid use was the most common tobacco form with male predominance [19]. A hospital-based cross-sectional study was conducted in Egypt, which reported that leukoplakia was the most frequently encountered lesion (3.54%), followed by oral lichen planus (2.88%) [20].

## Conclusions

Since the present study was an observational, cross-sectional study conducted on a small subset of the population, an elaborate cohort study with a large population is needed to conclude accurate data. Other predisposing predictors like nutritional and genetic status, alcohol synergism, BMI, and lifestyle, female patients with hesitant and incomplete history were not ruled out due to lack of periodic follow-up and lack of biopsy for confirming the OPMDs. Public awareness is the most important pillar regarding the benefits of early detection of OPMDs to avoid the occurrence of this disastrous oral cancer. To achieve this, a multifaceted approach that combines health education, regulation of government policies, bi-annual oral screening, and establishment of tobacco screening and cessation centers is highly advisable. The present study highlights the significance of the evidence and action-based approach toward society to control the deleterious effects of tobacco consumption and the significance of early detection, oral screening, and implementation of tobacco cessation counseling.

## Appendices

**Table 1:** STROBE (Strengthening the Reporting of Observational Studies) statement: checklist of items that should be included in reports of cross-sectional studies *Give information separately for exposed and unexposed groups.

	Item no.	Recommendation	Page no.
Title and abstract	1	(a) Indicate the study’s design with a commonly used term in the title or the abstract.	1
		(b) Provide in the abstract an informative and balanced summary of what was done and what was found.	1
Introduction			
Background/rationale	2	Explain the scientific background and rationale for the investigation being reported.	2,3
Objectives	3	State specific objectives, including any prespecified hypotheses.	3
Methods			
Study design	4	Present key elements of study design early in the paper.	4
Setting	5	Describe the setting, locations, and relevant dates, including periods of recruitment, exposure, follow-up, and data collection.	4
Participants	6	(a) Give the eligibility criteria and the sources and methods of selection of participants.	4
Variables	7	Clearly define all outcomes, exposures, predictors, potential confounders, and effect modifiers. Give diagnostic criteria, if applicable.	4
Data sources/ measurement	8*	For each variable of interest, give sources of data and details of methods of assessment (measurement). Describe comparability of assessment methods if there is more than one group.	4
Bias	9	Describe any efforts to address potential sources of bias.	4
Study size	10	Explain how the study size was arrived at.	4
Quantitative variables	11	Explain how quantitative variables were handled in the analyses. If applicable, describe which groupings were chosen and why.	4
Statistical methods	12	(a) Describe all statistical methods, including those used to control for confounding.	4
		(b) Describe any methods used to examine subgroups and interactions.	4
		(c) Explain how missing data were addressed.	Not applicable
		(d) If applicable, describe analytical methods taking account of sampling strategy.	Not applicable
		(e) Describe any sensitivity analyses.	Not applicable
Results			
Participants	13*	(a) Report numbers of individuals at each stage of study, e.g., numbers potentially eligible, examined for eligibility, confirmed eligible, included in the study, completing follow-up, and analyzed.	5
		(b) Give reasons for non-participation at each stage.	Not applicable
		(c) Consider use of a flow diagram.	Not applicable
Descriptive data	14*	(a) Give characteristics of study participants (e.g., demographic, clinical, social) and information on exposures and potential confounders.	5
		(b) Indicate number of participants with missing data for each variable of interest.	Not applicable
Outcome data	15*	Report numbers of outcome events or summary measures	
Main results	16	(a) Give unadjusted estimates and, if applicable, confounder-adjusted estimates and their precision (e.g., 95% confidence interval). Make clear which confounders were adjusted for and why they were included.	Not applicable
		(b) Report category boundaries when continuous variables were categorized.	5
		(c) If relevant, consider translating estimates of relative risk into absolute risk for a meaningful time period	Not applicable
Other analyses	17	Report other analyses done, e.g., analyses of subgroups and interactions, and sensitivity analyses.	Not applicable
Discussion			
Key results	18	Summarize key results with reference to study objectives.	6
Limitations	19	Discuss limitations of the study, taking into account sources of potential bias or imprecision. Discuss both direction and magnitude of any potential bias.	8,9
Interpretation	20	Give a cautious overall interpretation of results considering objectives, limitations, multiplicity of analyses, results from similar studies, and other relevant evidence.	6,7,8,9
Generalizability	21	Discuss the generalizability (external validity) of the study results.	9
Other information			
Funding	22	Give the source of funding and the role of the funders for the present study and, if applicable, for the original study on which the present article is based	Not applicable
